# P-1550. Systematic Literature Review of Acute Cystitis or Afebrile Urinary Tract Infection in Men

**DOI:** 10.1093/ofid/ofae631.1717

**Published:** 2025-01-29

**Authors:** Fanny S Mitrani-Gold, Amber Martin, Priscilla Wittkopf, Joanna Kamar, Madison T Preib, Ashish V Joshi, Aruni Mulgirigama

**Affiliations:** GlaxoSmithKline plc., Collegeville, Pennsylvania; Evidera, Bethesda, MD, USA, Waltham, MA; PPD Evidera, London, England, United Kingdom; PPD Evidera, London, England, United Kingdom; GSK, Collegeville, Pennsylvania; GlaxoSmithKline plc., Collegeville, Pennsylvania; GlaxoSmithKline plc., Collegeville, Pennsylvania

## Abstract

**Background:**

Acute cystitis in men is classified as complicated urinary tract infection (UTI), despite the absence of functional and/or structural anatomical abnormalities of the urinary tract, associated with a higher risk of treatment failure. We conducted a systematic literature review (SLR) to identify and summarize evidence on the epidemiology (incidence, etiology, antimicrobial resistance [AMR], recurrence) and treatment patterns of acute cystitis or afebrile UTI among men with no signs of systemic infection.

**Figure:**
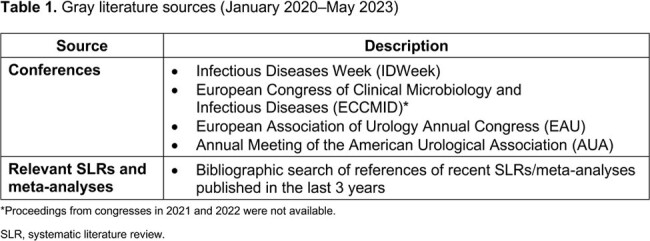

**Methods:**

Systematic searches for English language articles were conducted Jan 1, 2011–May 12, 2023 in the MEDLINE, MEDLINE In-Process, and Embase databases via OvidSP. Gray literature sources (**Table 1**) were reviewed. Publications were evaluated by 2 independent reviewers (**Table 2**).

**Figure:**
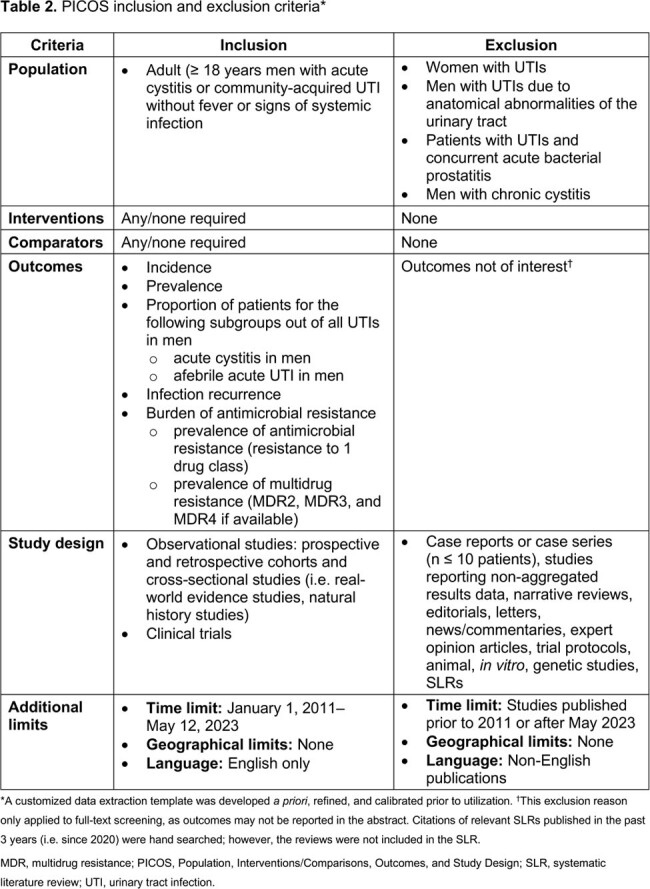

**Results:**

Database searches yielded 1025 records with 6 studies eligible for inclusion (**Figure 1**). Studies were primarily excluded if the population of interest was not included or if outcomes were inseparable for the population of interest. The 6 studies were conducted 2012–2020 in 6 geographic regions (1 US). Sample sizes ranged from 18 to 4876 patients. Four studies reported age (median 44–71 years; **Table 3**). Population of interest was defined as “uncomplicated” (3 studies) or “acute cystitis”/“afebrile UTI” (3 studies). The incidence of uncomplicated UTI was 6.5/1000 person-years in a retrospective cohort (Netherlands) and increased with age. Relapse, defined as continuation of UTI symptoms ≤ 28 days of stopping treatment, after initial symptom resolution, ranged from 9.9% to 16.9% (referred to as recurrence rates in this study). *Escherichia coli* (*E. coli*) were the etiologic bacteria in 39–94% of patients. Nitrofurantoin (56.0%) and fluoroquinolones (62.8%) were the most commonly prescribed antibiotics in the Netherlands and Japan, respectively. AMR among *E. coli* ranged from 11.8% to 47.1% in 1 study.

**Figure:**
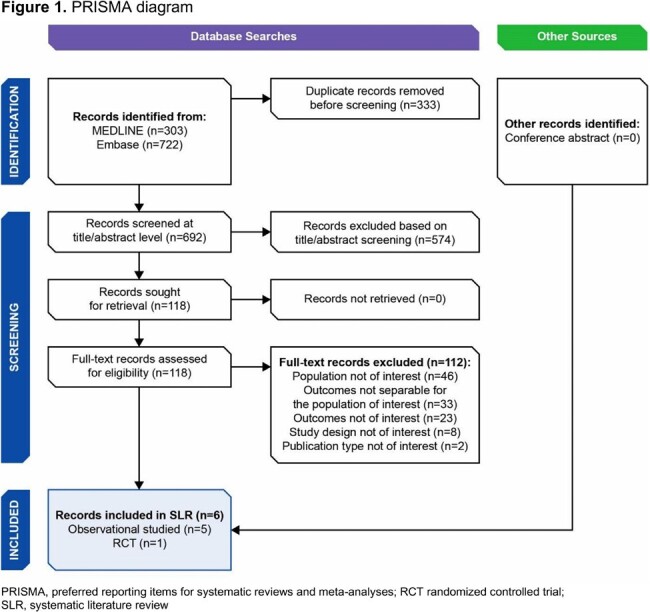

**Conclusion:**

AMR in males with acute cystitis/afebrile UTI may be higher than females in some geographic regions. The SLR identified 6 studies with heterogenous designs, inclusion criteria, and UTI definitions, highlighting the need for research using standard definitions to characterize acute cystitis/afebrile UTI in men, including recurrence rates and the burden of AMR.

**Funding:** GSK study 220916

**Figure:**
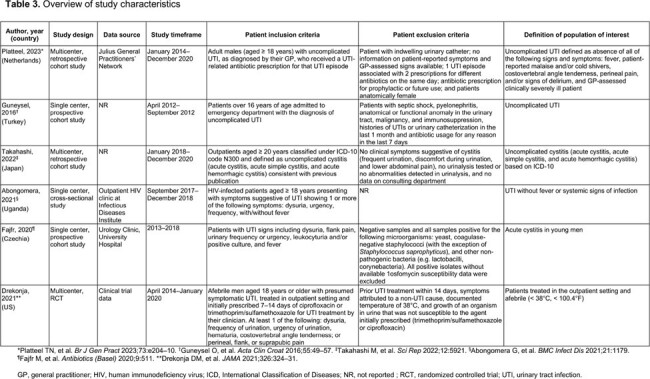

**Disclosures:**

**Fanny S. Mitrani-Gold, MPH**, GSK: Employee|GSK: Stocks/Bonds (Public Company) **Amber Martin, BS**, PPD Evidera: Employee of PPD Evidera, which received funding from GSK for this study. **Priscilla Wittkopf, PhD**, GSK: Grant/Research Support|PPD Evidera: Employee of PPD Evidera, which received funding from GSK for this study.|ThermoFisher Scientific: Stocks/Bonds (Private Company) **Joanna Kamar, MPH**, PPD Evidera: Employee of PPD Evidera, which received funding from GSK for this study. **Madison T. Preib, MPH**, GSK: Employee|GSK: Stocks/Bonds (Public Company) **Ashish V. Joshi, PhD**, GSK: Employee|GSK: Stocks/Bonds (Public Company) **Aruni Mulgirigama, MBBS**, GSK: Employee|GSK: Stocks/Bonds (Public Company)

